# Goal Commitments and the content of thoughts and dreams: basic principles

**DOI:** 10.3389/fpsyg.2013.00415

**Published:** 2013-07-11

**Authors:** Eric Klinger

**Affiliations:** Psychology Discipline, Division of Social Sciences, University of MinnesotaMorris, MN, USA

**Keywords:** mind-wandering, goals, dreams, default-mode network, planning, creativity, memory, rumination

## Abstract

A few empirically supported principles can account for much of the thematic content of waking thought, including rumination, and dreams. (1) An individual’s commitments to particular goals sensitize the individual to respond to cues associated with those goals. The cues may be external or internal in the person’s own mental activity. The responses may take the form of noticing the cues, storing them in memory, having thoughts or dream segments related to them, and/or taking action. Noticing may be conscious or not. Goals may be any desired endpoint of a behavioral sequence, including finding out more about something, i.e., exploring possible goals, such as job possibilities or personal relationships. (2) Such responses are accompanied and perhaps preceded by protoemotional activity or full emotional arousal, the amplitude of which determines the likelihood of response and is related to the value placed on the goal. (3) When the individual is in a situation conducive to making progress toward attaining the goal, the response to goal cues takes the form of actions or operant mental acts that advance the goal pursuit. (4) When circumstances are unfavorable for goal-directed operant behavior, the response remains purely mental, as in mind-wandering and dreaming, but still reflects the content of the goal pursuit or associated content. (5) Respondent responses such as mind-wandering are more likely when the individual is mentally unoccupied with ongoing tasks and less likely the more that is at stake in the ongoing task. The probability of respondent thought is highest during relaxed periods, when the brain’s default-mode network dominates, or during sleep. The article briefly summarizes neurocognitive findings that relate to mind-wandering and evidence regarding adverse effects of mind-wandering on task performance as well as evidence suggesting adaptive functions in regard to creative problem-solving, planning, resisting delay discounting, and memory consolidation.

## INTRODUCTION

The basic thesis of this article is that the thematic content of thoughts and dreams is determined, directly or indirectly, by the individual’s goals. This article answers, as far as the current state of research permits, the question of how we may predict the thematic content of what individuals will think or dream about at any given moment in time, or how much they will think about something over longer time periods. Will it be, for instance, about a particular personal relationship, a problem at work, a religious quandary, a political situation, fear of leading a life of failure, or competing for a prize? Why will the mix be different for Person A than for Person B? When is the individual most likely to think about a particular topic? If the thought is in the form of mind-wandering while engaged in a task, to what extent is this a waste of time?

### HISTORY AND SOME DEFINITIONS

Thematic content simply refers here to what the thought or dream is about, irrespective of the form in which it occurs, whether verbal or non-verbal, conceptual or imaginal, whether its representation of the content is fairly veridical, metaphoric, symbolic, or associated with the thematic content in some other discernible way. These latter dimensions of form raise a host of other questions, the answers to which have been little researched, are not as well understood, and are not addressed in this article.

While people are working on specific tasks, much of their mental content will, of course, relate to the momentary task. However, even during such *operant* task activity, mental content commonly shifts intermittently to other, *respondent* content (Klinger, 1971; Andrews-Hanna et al., 2010a), which has often been dubbed daydreaming or mind-wandering.

Modern scientific research on such shifts in thought can be considered to have begun in earnest with the experimental and psychometric work of Jerome L. Singer and John S. Antrobus (e.g., Singer, 1966). The terms operant versus respondent are taken from Skinner’s theory (Skinner, 1935, 1953) to refer to activity aimed at acquiring reinforcers (or goals) versus reflexive responses to stimuli, here including one’s own mental events.

Various investigators have applied varying operational definitions to daydreaming, such as thought consisting of fanciful content (Freud, 1953), being independent of current task activity (e.g., Singer, 1966), or being unintended and spontaneous, i.e., *respondent* (Klinger, 1971). As it turns out, when assessed by thought-sampling with participants’ self-reports and analyzed intra-individually, these three definitions are almost orthogonal (Klinger and Cox, 1987–1988). That is, where a thought scores on any one of these dimensions provides almost no information as to where it will score on either of the other two dimensions.

The easiest of these thought dimensions to operationalize is independence from current tasks, also dubbed mind-wandering, which constitutes between about a third and a half of waking thoughts (Klinger and Cox, 1987–1988; Andrews-Hanna et al., 2010a; Killingsworth and Gilbert, 2010). Mind-wandering has in recent years attracted strong interest among cognitive and neurocognitive researchers. Their work has greatly enriched knowledge of mind-wandering.

### EVOLUTIONARY CONSIDERATIONS: WHAT GOOD IS MIND-WANDERING?

What follows is shaped by two evolutionary considerations. *First*, any kind of activity that absorbs up to a half of conscious brain activity must have been selected for its contribution to the human species’ survival. Indeed, it appears that the brain’s *default-mode network* provides the substrate for mind-wandering (e.g., Mason et al., 2007; Christoff et al., 2009; Andrews-Hanna et al., 2010a; Stawarczyk et al., 2011b), a network of several “hubs” and “subsystems” (Andrews-Hanna, 2012) that probably constitutes a majority of the brain’s energy consumption (Raichle, 2009). It must serve important functions. These include a variety of mental processes, including retrieval of past experiences and imagining future scenarios (Buckner et al., 2008), which are essential for planning and are also stock components of mind-wandering sequences.

Raichle (2009) argues that the default-mode network is not coextensive with conscious mind-wandering, citing the continuation of the network’s activity into lighter states of anesthesia and Stages 1 and 2 of sleep (Horovitz et al., 2008), when dreaming is most frequent, and the demonstration (Christoff et al., 2009) of executive-network elements during resting states. However, if one accepts that there is continuity between mind-wandering states and dreaming, and if one recognizes that non-conscious processes (*meaning-complexes*; Klinger, 1971, 2011) underlie both the thought and dream segments, there is no reason to doubt the close relationship of mind-wandering and its variants with the default-mode network.

There is increasing reason to believe that spontaneous, respondent thought, as in mind-wandering and other forms of daydreaming, is continuous with night-dreaming, their different properties being attributable to the different neurophysiological contexts in which they operate – that is, a single round-the-clock stream of mentation modulated by fluctuations of brain states but without sharp disjunctions in its phenomenology. There are dream-like segments in waking states – in one study 25% of waking thought samples were rated by participants as having at least a trace of dream-like qualities (Klinger and Cox, 1987–1988), which agrees approximately with other results (Foulkes and Fleisher, 1975; Klinger, 1978-79) – as well as there being waking-like cognitive content in dreams. As indicated below, goal-related cues have somewhat similar effects on the content of both waking thought samples and dream samples obtained with night-time probes. Some degree of continuity in waking and sleeping mentation has been noted as well by others (e.g., Domhoff, 1996; Raichle, 2009; Christoff et al., 2011). Given this apparent continuity, it seems reasonable to consider both kinds of states together for purposes of delineating principles governing their thematic content.

A *second *evolutionary consideration has to do with the fact that succeeding in goal pursuits is, for the Animal Kingdom, a categorical imperative. That is, all animal species at one or more of their life stages depend for their individual survival on successful pursuit of the substances and conditions necessary for life – a motile survival strategy. Furthermore, the survival of the species depends on individuals successfully pursuing the goals necessary for procreation.

It follows that every adaptation conferred on humans by the natural selection that produces evolution must have evolved in the service, direct or indirect, of successful goal pursuit. That would include respondent mental activity, such as mind-wandering. These two evolutionary considerations provide a backdrop for the following five principles that account for much of the variance in the thematic content of people’s thoughts.

(A definitional clarification: Goals may be any targeted endpoint of a behavioral sequence, such as food, sex, sleep, a paycheck, an aesthetic experience, or finding out more about something, i.e., exploring *potential* goals, such as job possibilities or personal relationships. The brain’s processes underlying goal pursuit fit the conventional definition of *motivation*.)

## PRINCIPLE 1: GOAL COMMITMENTS DETERMINE ATTENTIONAL FOCUS, RECALL, THOUGHT AND DREAM CONTENT, AND BEHAVIORAL FOLLOW-THROUGH

An individual’s commitments to particular goals sensitize the individual to respond to cues associated with those goals. These cues then receive automatic priority for cognitive processing. The cues may be external in the environment or internal in the person’s own mental activity and include cues related to failure to achieve a goal (Chatard and Selimbegović, 2011, Study 6). The responses may take the form of noticing the cues, storing them in memory, having thoughts or dream segments related to them, and/or taking action. Noticing may be conscious or not.

One’s personal goals also provide an important basis for organizing one’s thoughts, including thoughts about one’s future (*prospection*), and introducing goal-related cues appears to facilitate this process (D’Argembeau and Demblon, 2012). People are also more fluent in making up scenarios around specific goals than around specific persons or locations (D’Argembeau and Mathy, 2011). These investigators, however, instructed their participants to formulate scenarios about hypothetical future events. The responses were not primarily the mind-wandering – spontaneous, undirected thoughts – on which the remainder of this article will focus.

### EFFECTS OF GOALS ON THOUGHT AND DREAM CONTENT

Early support for this view (Klinger, 1978; Hoelscher et al., 1981) demonstrated effects of goals on attention, recall, thought content, and dream content. Initial investigations of this model assessed participants’ concerns (the state of having goals, both positive-appetitive and aversive, as assessed there from extensive interviews and questionnaires) and a few days later asked participants to listen with moderate attention to what they were about to hear. The experimenters then played for them simultaneously two different but similar audiotaped 15-min narratives, one narrative to each ear.

Throughout, participants could choose to which narrative they listened. Here and there, at particular time points on this tape, a few words going to one ear had been inconspicuously modified to relate to one of the individual participant’s own goals, and, simultaneously, a few words going to the other ear had been modified to relate to someone else’s goals, but not, so far as one could determine, to this participant’s goals. The two modified passages in each such pair were matched on formal properties and designed to be syntactically compatible with the words they replaced. For example, for a participant who had the goal of entering a helping profession, the italicized words in the following passage were embedded into one of the narratives (original stream-of-consciousness fiction, *Texts for Nothing*, by Beckett, 1995, p. 101): “Who are these people anyway? *What do they need, what can be given?* Did they follow me up here...” The narrative passages in which the modified portion related to the participant versus to someone else varied quasi-randomly between the two narratives within each session.

Participants used a toggle switch to indicate points in time at which they switched the ear to which they were paying attention. This indicated at any given time to which narrative they were probably listening. Ten seconds after the end of each modified passage, the tape paused with a signal tone and participants reported what they were thinking about and what last content they recalled from the tape.

The results were powerful (**Table 1**): participants spent significantly more time listening to passages associated with their own goals than to passages associated with others’ goals, recalled those passages about twice as often, and had thought content that (by ratings of judges blind to conditions) was related to passages associated with their own goals about twice as often as to the opposite passages (Klinger, 1978). The relatively small differences in time spent attending to the own-goal-related cues versus other cues is explainable by the fact that detecting such cues in an unattended channel requires the process of noticing them after a portion of the own-goal-related passage has already played, then switching attention to that channel and moving the toggle switch.

**Table 1:** Cognitive responsiveness to concern-related versus non-concern-related cues.

Concern	Types of cues		Percent of intra-subject variance accounted for by cue differences
	Concern	Non-concern
**Waking subjects**
Time spent listening (seconds)	74.6	58.9^b^	7.62
No. of passages per session recalled	2.78	l.38^c^	17.06
Passages per session thought about	3.73	1.95^c^	18.59
**Sleeping subjects**
Percent cues incorporated in	34	11^a^
REM sleep		

*Note. Data for waking subjects are from Klinger (1978), Table V, p. 252. Significance values are for the differences between concern-related and non-concern-related means. The differences were tested by directional *t*-tests for correlated data, with each session’s mean difference between Concern and Non-concern conditions taken as the unit of analysis, adjusted to compensate for differing numbers of sessions per participant. The data are based on 68 sessions from 20 participants (17 for percent of variance), but because of a Behrens–Fisher problem degrees of freedom used in significance testing were the most conservative number, 19 d.f. Data in the bottom row from seven sleeping participants for 59 stimulus trials during REM over three experimental nights are from Hoelscher et al. (1981). REM, rapid eye movement sleep.*

a *p *< 0.05

b *p *< 0.0005

c *p *< 0.0001.

When participants listened to tapes that had been prepared for other participants, no effects on attention, recall, or thought content occurred, indicating that the embedded cues indeed corresponded to the goals of the individual participants. The fact that the cues were woven unobtrusively into their narrative contexts, with no particular tasks to be performed with them, suggests that they might have functioned in a way similar to unexpected environmental cues or even cues presented by the individual’s own internal stream of consciousness.

Similarly, goal-related stimuli influence dream content much more reliably than do other stimuli. One investigation in a sleep laboratory using standard electroencephalography (EEG) and eye movement measures (Hoelscher et al., 1981) administered a modified Concern Dimensions Questionnaire (CDQ; Klinger et al., 1980, 1981) to assess seven participants’ goals, followed by four consecutive nights, an adaptation night and three experimental nights, during which, five to seven times per night during Stage 1-rapid eye movement (REM) or Stage 2 sleep, the experimenters played for them various taped words or phrases related to their different goals or to other participants’ goals. Eight seconds after each such stimulation, participants were awakened to orally report their dream content, which was recorded. Judges blind to condition scored the reports for three types of thematic incorporation: (a) direct mention of the stimulus, (b) mention by synonymous language, and (c) metaphoric parallels or thematic similarities to the CDQ descriptions of the concern or non-concern represented by the stimulus. Pairs of raters agreed on incorporation judgments 94% of the time, compared to a chance agreement level of 67% (*F*(73) = 3.42, *p < *0.005). Dream reports resembled the immediately preceding stimuli about three times as often if the stimuli related to participants’ own goals than if they related to other people’s goals (**Table 1**). These results are consistent with conclusions drawn from an extensive review of the dream literature by Domhoff (1996).

In these studies, comparing thoughts and dreams to stimuli played after the thoughts and dreams had already occurred produced little resemblance, regardless of how goal-related the stimulus was. Thus, the effect on thought and dream content was clearly produced by the goal-relatedness of the stimuli that preceded the thoughts and dreams. *That is, the state of having a goal* – *i.e., a current concern about that goal* – *automatically gave processing* priority in the individual’s cognitive systems to cues associated with that goal.

Another sleep study (Nikles et al., 1998) instructed 10 participants before they went to sleep to dream about a particular topic, which sometimes was related to one of their individual goals, as assessed by a modified Short Motivational Structure Questionnaire (MSQ; Cox and Klinger, 2011) and sometimes it was related to a different person’s goal. Participants spent four consecutive nights in the laboratory: an adaptation night without awakenings, a baseline night with awakenings but no dream suggestions, and two experimental nights with the presleep suggestions. Half of the participants received own-goal instructions on Night 3 and instructions to dream about another’s goal on Night 4, with the other half receiving the reverse order of instructions. The instructions to dream about topics that were related to participants’ own goals significantly influenced dream content (as rated by judges blind to conditions), whereas instructions to dream about topics related to others’ goals did not. These results indicated that suggestions to dream about their own goals engaged participants’ attention, recall, and subsequent dream processing in a way not found with suggestions to dream about other topics.

Additionally, although the rate at which own-goal-related material appeared in dreams was lower during the baseline night, when no dream topics had been suggested, than when they had been suggested on an experimental night, dreaming related to own goals was still significantly more frequent (*p* < 0.01) than material related to others’ goals, which rarely occurred. This parallels the findings obtained with goal effects on waking thoughts, such as in mind-wandering. The processing priority conferred by having a goal – a current concern – continues to operate in dreams during sleep.

The results of the suggestion nights also demonstrated that dream content responded to sleepers’ presleep intentions, which would appear difficult for activation-synthesis theory to accommodate. The principal features of Hobson and McCarley’s (1977) original formulation of activation-synthesis theory are that (a) dream images are directly generated by essentially random, sporadic pontine discharges (the activation) that produce sudden sensations, the dream hallucinations, and (b) forebrain activity seeks to fit these images into as coherent a pattern or plot as possible (the synthesis). There is also an extended version of this theory (Seligman and Yellen, 1987) that adds an emotional component. Although one could argue that goal-related associations might be worked into the synthesis, neither formulation admits volitional forces such as intentions into the array of determinants that shape dreams, let alone an interaction of intentions with current concerns.

Taken together, the findings described in this section indicate the powerful effect that goal-related cues exert on thought flow in states that cannot be considered conducive to goal-directed operant activity. The cues used in experiments with waking participants (Klinger, 1978) were subtly embedded in spoken narratives to which participants were simply instructed to listen with moderate attention. For example, for a participant one of whose goals was to enter a helping profession, an allusion to someone’s needs was embedded in an otherwise irrelevant 15-min narrative played to one ear, while simultaneously a cue considered unrelated to the participant’s goals was embedded in another narrative played to the other ear. Such cues could not have been interpreted as discriminative stimuli for action. Similarly, the dream study by Hoelscher et al. (1981) exposed certifiably sleeping participants to cues relating to their goals, such as an experimenter speaking the address of a boyfriend. The participants remained asleep until awakened by the experimenters for their dream reports. These results identify goal-related stimuli as one of the triggers for individual mind-wandering and dream segments.

It is a matter of definition as to whether such segments could be considered self-generated, given that the cues were external. However, internal cues from a person’s previous stream of mentation might plausibly play a role similar to the external cues employed in the above experiments. Insofar as that is true, it answers provisionally one of the questions raised by Smallwood (2013a) regarding the determinants of when (“why”) self-generated thoughts begin: exposure to goal-related cues.

The thoughts evoked by cues and those occurring naturally do, of course, eventually end, and rather soon. Nobody has so far devised a satisfactory method for assessing when naturally occurring thought segments begin and end. The only method so far available is to rely on participants’ retrospective self-reports, which are bound to be fraught with error. However, a group of 20 participants trained to estimate brief time lapses rated the durations of the latest thought segments prior to probes, and of the segments just preceding those, in both laboratory settings and, for 12 of them, while living their otherwise normal daily lives. Their median estimates of segment duration were 5 s in both settings, with a mean of 9 s in the laboratory setting and 14 s outside the laboratory (Klinger, 1978). These participants rated their confidence in their own estimates as “very confident” 64% of the time and as “moderately confident” 35% of the time. Pope (1977) asked participants in a laboratory to signal with a key press every time their mind shifted to a new topic, which happened on average about 5 or 6 s apart. This agrees very approximately with our own findings.

The implication is that mental content continually jumps from goal-related topic to goal-related topic in brief segments that may or may not return to the same topic as previous segments. A very rough estimate provides the generalization that waking mental activity over a 16-h day contains about 4,000 such thought segments (Klinger, 1990).

### INTERFERENCE STUDIES OF AUTOMATIC PROCESSING PRIORITY FOR GOAL-RELATED CUES

The processing priority for goal-related cues has been shown using a variety of other cognitive methods. One is the use of quasi-Stroop procedures. In the classic Stroop procedure, under instructions to name the color of the font of words displayed one at a time as quickly as possible, participants typically respond more slowly when the meaning of the word conflicts with the color, such as green font for the word RED. Similarly, reaction times (RTs) in reporting the font color of goal-related words are typically on average longer than they are to non-goal-related words (Johnsen et al., 1994; Riemann and McNally, 1995; Gilboa-Schechtman et al., 2000; Fadardi and Cox, 2008) or images. Presumably, the own-goal-relatedness of the word’s meaning grabs processing priority over identification of font color, thereby slowing reporting of font color. This processing priority could readily account for the tendency of conscious mental content in mind-wandering to gravitate toward material related to the individual’s own goals. The stimuli for such shifts in content are presumably internal ones in the individual’s own ongoing stream of thought.

W. Miles Cox and his colleagues have extended this work in a variety of ways to consumers of alcohol. Recalling that goals influence the focus of attention, it is no surprise that excessive drinkers find their attention drawn to alcohol cues. Instead of targeting RT to words whose meaning conflicts with their font color, these studies targeted words related to alcohol and compared RT to the alcohol-related words with RT to emotionally neutral words (Cox et al., 2000, 2006). Frequent drinkers were slower to name the colors of alcohol-related words, and the amount of interference, which is calculated as RT to alcohol words *minus *RT to neutral words, is correlated with the amount of alcohol the participant normally consumed in a week (Fadardi and Cox, 2008, 2009; This effect was largely independent of participants’ overall executive cognitive functioning).

This attentional bias is problematic for people who wish to reduce their drinking (Cox et al., 2002; Fadardi and Cox, 2008), at least partly because drink cues arouse the desire to drink. Interestingly, Fadardi and Cox (2009) have worked out an intervention, called the Alcohol Attention Control Treatment Program (AACTP), for reducing excessive drinkers’ bias toward processing alcohol cues. In this method, they attempted to counter the processing priority of alcohol cues directly by training disattention to them in successive Stroop exercises. The method both improved color-naming RT and reduced participants’ alcohol consumption. The reduction of alcohol consumption continued through a 30-day follow-up period. It was evidently possible to reduce the processing priority of the drinking goal and with it the status of the goal itself.

The automatic character of this cognitive prioritizing was further buttressed by data from a lexical decision task (Young, 1987). Young’s participants were to indicate as quickly as possible by pressing a button whether each occurrence of a letter string on a computer screen was an English word. The left side of the screen was taken up by a patch containing computer-related verbal “garbage,” which participants were instructed to ignore (and apparently did), but which sometimes contained a word related to one of a participant’s current goals. When the target string was indeed a word, this lexical judgment was slowed significantly if the distractor patch contained an own-goal-related word. Again, the point here is that encountering cues related in some way to one’s goals takes higher processing priority over competing cues in a way that helps to explain the gravitation of undirected thought content to one or another of the individual’s goals.

On the assumption that males are more likely than women to be concerned with power and hence be drawn to its cues, Mason et al. (2010) performed three experiments on responses of men and women to high-power versus low-power cues. These demonstrated that male participants dwelt longer on words relevant to power than on neutral words, were more distracted by high-power than low-power flanker words (i.e., had longer RTs to target stimuli), and were more likely to recall high-power-related than low-power-related names. Female participants did not display these biases of attention and memory. These results are consistent with those described above. Goal-related cues outrank others in processing priority.

### BRAIN FINDINGS RELEVANT TO GOAL EFFECTS ON COGNITIVE PROCESSING

Recent brain-imaging studies have provided further support for this conclusion. Ihssen et al. (2011) displayed single alcoholic beverages and four other kinds of images while assessing brain activity with magnetic resonance imaging (MRI) in light and heavy drinkers. In contrast to the light drinkers, the heavy drinkers responded in key cortical areas (right and left insula and ventral striatum) significantly more strongly than in response to neutral stimuli. This is consistent with the behavioral evidence that alcohol cues strongly draw the attention of heavy drinkers, for whom drinking is a frequent goal.

Consistent with these findings regarding the processing priority placed on cues related to goals, Franz (2012) has presented a neuroscientific model of brain organization and development, based largely on split-brain research, to support the conclusion that attention is controlled by intended actions and, at higher levels of organization, goals. He proposes “a multilevel system for the allocation of attention for action, in which the dopaminergic basal ganglia-thalamic-cortical circuits are integral.... Notably, the present framework builds upon a highly dynamic system in which subcortical processes are central to the networks involved” (p. 12). Attention is thereby tethered to the brain systems responsible for central motivational processes.

An impressive proportion of thought samples obtained during mind-wandering have contained content that related to the individual’s goals, including spontaneous planning elements (Andrews-Hanna et al., 2010a; Stawarczyk et al., 2011a; Andrews-Hanna, 2012). In a retrospective questionnaire following their MRI sessions, participants using seven-point scales rated their spontaneous thoughts on average at about a goal-relatedness scale’s midpoint (4.16) and rated the thoughts’ personal significance above that (5.26); nearly half of these thoughts were considered to have focused on the past (19%) or future (28%; Andrews-Hanna et al., 2010a). Their *f*MRI-based brain activity correlations indicated that besides the more established components of the default-mode network, such as the posterior cingulate cortex and medial prefrontal cortex, the medial temporal lobe plays an important role in supplying material for spontaneous thoughts from the individual’s past or prospective future (Andrews-Hanna et al., 2010a,b). Consistent with these findings, Ellamil et al. (2012) found elevated activity in the medial temporal lobes during generation of creative ideas.

While arguing vigorously for the adaptive nature of the default-mode network, Schacter (2012) observed that “in most studies that have linked default network activity with simulation of future experiences, the simulated future events are not linked to formulating a plan, solving a future problem, or any other kind of goal-directed cognitive activity. Instead, they represent imaginary scenes or scenarios that might or might not occur to the individual within a particular future time frame.” Unorganized as they may be, they serve up insights and planning components that can be assembled later into adaptive plans and action, as suggested by the incubation effect of experimentally induced mind-wandering in improving creative problem-solving (Baird et al., 2012). See also below under Principle 4.

Christoff et al. (2009) (see also Christoff, 2012 for elaboration) discovered that mind-wandering often includes an interweaving of default-network activity with executive systems. One of the latter is the frontoparietal control network, which appears to couple with the default-mode network during planning activity (Spreng et al., 2010). This is consistent with the findings of autobiographic planning during mind-wandering (Baird et al., 2011; Stawarczyk et al., 2011a) – presumably planning for action on one of the participant’s goals.

## PRINCIPLE 2: EMOTIONAL RESPONSES PROBABLY PLAY A ROLE IN INITIATING AND ACCOMPANYING ATTENTION TO GOAL-RELATED CUES

There are a number of reasons for believing that responses to goal-related cues are accompanied and perhaps preceded by protoemotional activity or full emotional arousal, the amplitude of which determines the likelihood of response and is related to the value placed on the goal. First, ratings of a cue’s goal-relatedness are strongly correlated with ratings of emotional responses to those cues. Second, goal-related cues arouse skin conductance responses more dependably than do other cues, a measure of arousal. Finally, anticipated emotional responses to reaching a goal or to failure to reach it are a measure of the goal’s value to the individual.

As a terminological note, “arousal” is here taken as a dimension of emotional amplitude. From at least Wilhelm Wundt a century ago to Lisa Feldman Barrett (e.g., Kuppens et al., 2012), arousal (sometimes called activation), and hedonic valence (i.e., quality of the emotional response being aroused) have been two fundamental dimensions of emotion or affect. Sometimes, as in the literature on skin conductance responses, the hedonic valence of the aroused emotion is left unspecified.

### CORRELATIONS BETWEEN EMOTIONAL EVOCATIVENESS AND GOAL-RELATEDNESS OF CUES

Regarding the association of emotional arousal with goal-related cues, Bock and Klinger (1986) computed 85 participants’ intra-individual correlations between two kinds of their reactions to 40 words: the word’s emotional “arousal potential” for them (“the strength of the subject’s emotional reaction to the content of the word”) and “the extent to which the word has to do with the subject’s important concerns, problems, worries, or goals that currently preoccupy the subject.” The two ratings were obtained in two different phases of the experiment, separated by a distractor task. The mean intra-individual correlation between these ratings was 0.45 (*p* < 0.0001). Partial replications of this relationship in unpublished data (E. Klinger, S. J. Perrine, E. S. Goetzman, T. Hughes, M. Bock, U. Bowi) yielded similar intra-individual coefficients of 0.57, 0.63, 0.65, and 0.60 (all significant at *p* < 0.001).

### AN INTERFERENCE STUDY

Using RT methods (Schneider, 1987), participants pressed buttons as quickly as possible to identify whether letters displayed one at a time on the lower half of a computer screen were an X or a Y. Participants were instructed to ignore distractor stimuli, many of them words, that often appeared at a fixation point above the letters. After this procedure, subjects rated how much each distractor word aroused them emotionally. Letter identification slowed significantly when the distractor words were rated as emotionally arousing. That this effect really was attributable to something emotional is supported by the fact that participants who scored high on the Affective Intensity Measure (Larsen and Diener, 1987) were slowed by emotionally arousing distractors significantly more than other participants were.

### SKIN CONDUCTANCE RESPONSES TO GOAL-RELATED CUES AND ASSOCIATION WITH GOAL-RELATED THOUGHTS

Additional data on the relation of emotional arousal to goal cues were obtained with skin conductance responses (Nikula et al., 1993). In the first of three experiments, 19 American participants participated in two separate sessions. The first session consisted of completing the CDQ (Klinger et al., 1980), and the second session on the following day involved electrodermal measurement during taped stimulus presentations. These consisted of 84 three-word clusters presented twice in two different random orders; for example, “doctor lifelong ambition,” “roommate threatens existence,” “student journal editor,” or “acquire darkened appearance.” Eight of these, *concern clusters*, were constructed separately for each participant based on his or her CDQ responses, in most cases masked by avoiding using participants’ own wording. Four other word clusters were drawn from a pool of other participants’ concerns with verification that they were not of concern to the particular participant, and 72 were filler clusters constructed to be formally like the concern clusters. The mean range-corrected proportional increase in skin conductance for intervals extending to 5 s after the end of stimulus clusters was 0.525 for concern clusters, 0.273 for non-concern clusters, and 0.174 for filler clusters. The difference between concern and non-concern clusters was significant with *p* *< *0.025 and a Cohen effect size of 0.56. The difference in skin conductance between filler clusters and concern clusters was significant with *p* *< *0.01.

Unfortunately, the experimenters subjectively suspected that 8 of the 19 Minnesota participants may have had at least some inkling of the hypotheses of this study, although this was not confirmed by participants’ statements. In any event, the skin conductance results for the 11 probably unaware participants were weaker than for the possibly aware participants and, taken by themselves, fell short of statistical significance (*p < *0.10, two-tailed). This made it desirable to attempt a different kind of research design using a thought-sampling procedure. Instead of assessing arousal in response to goal-related and neutral cues constructed by the investigators (which participants may have recognized correctly as having been constructed for them individually), Experiments 2 and 3 investigated what was happening in participants’ consciousness as a function of skin conductance measures. In Experiments 2 (158 German students) and 3 (24 German students), the signal tones to rate thoughts during electrodermal measurement were sounded either when experimenters observed non-specific (i.e., unelicited) skin conductance responses (10 probes) or at control points (also 10 probes) in the absence of such spontaneous electrodermal activity, in quasi-random order. Experiments 2 and 3 differed only in the larger number of scales in Experiment 3 on which participants orally rated their thoughts at each sampling point. In Experiment 2, they rated the presence of a current concern (unfinished “activity or goal”), arousal, and imagery (“imagination”) in their thought content. These ratings were on a scale ranging from 0 (not present) to 5 (fully present).

The mean ratings for current-concern-relatedness were 2.18 after probes triggered by skin conductance responses and 1.88 after probes during electrodermally inactive periods (*p* < 0.001). Ratings of perceived arousal differed much less (*p* < 0.05) between these two sampling conditions and of imagination not at all. In Experiment 3, the ratings that reached significance at *p* < 0.05 were higher after probes triggered by skin conductance responses than during quiescent periods for current-concern-relatedness and for anxiety, and they were lower for presence of imagery. Together, these findings indicate through electrodermal measures that internally generated goal-related cues are either preceded or closely accompanied by emotional arousal. That the difference in ratings of arousal was weaker than those of concern-relatedness may indicate that the arousal is often not consciously experienced.

Also of interest in Experiment 3 was the rating variable for “dormant concerns” (“any activity or goal you have not finished or reached in the past which you are not pursuing currently”). Insofar as ratings of the presence of dormant concerns in thought samples differed by sampling conditions (*p* < 0.10), they were actually lower during skin conductance responses than during quiescent periods. This suggests that *current* goal pursuit, rather than just having the goal in one’s past conceptual repertoire, is necessary for arousal.

### HEART RATE LEVELS AND THE FREQUENCY OF TASK-UNRELATED THOUGHTS

Another investigation (Smallwood et al., 2004) found evidence of substantially higher arousal as measured by heart rate for individuals who engaged in more frequent task-unrelated thoughts (TUTs). Although this investigation did not assess the relatedness of TUTs to individual participants’ goals or concerns, it is a fair assumption from other evidence that their TUTs were so related. Although the sample was small, the correlations between TUT frequency and heart rate were substantial. For three task conditions the ordinal correlations were 0.75 in a word-shadowing task (reading without memorization) and 0.28 in a word-study task (with memorization); the overall correlation for the combined tasks was 0.58. One possible implication is that goal-related thoughts during TUTs are associated with emotional arousal.

### EEG EVIDENCE ON LATENCY OF PROTOEMOTIONAL RESPONSES TO EMOTIONALLY EVOCATIVE STIMULI: IMPLICATIONS FOR CONTROL OF ATTENTION

Although the studies above demonstrate an association between emotional arousal and cues of one’s goals, they provide no evidence regarding the temporal sequence of this association. However, EEG evidence is at least suggestive, in the form of associations between responses to emotionally evocative stimuli – both words and pictures – and positive deflections in the EEG trace in a band beginning at about 300 ms after stimulus onset (the P300 response). This nomological net is reviewed elsewhere (Klinger, 1996). Its implications are that the processing of emotionally loaded material begins about a third of a second after stimulus onset. Because of its association with the emotionality of stimuli at a point when the arousal is not yet conscious, I have dubbed this kind of response *protoemotional*.

In this view, the protoemotional response constitutes a first step in processing of stimuli; whether processing continues, and whether it eventually engages other bodily systems, such as circulatory, glandular, pulmonary, or intestinal activity, depends on conditioned responses and cognitive assessments of the importance, valence, and expectancies of whatever it is that the stimuli represent. Insofar as emotionally evocative stimuli are also goal-related stimuli, having a goal – a current concern – controls much of what it is to which people attend.

### MOOD, MIND-WANDERING, AND RUMINATION

There is one more kind of association involving emotion. There is evidence that inducing negative moods increases mind-wandering, perhaps because it potentiates personal concerns (Smallwood et al., 2009a). When internal cues are associated with threat, they may set up a repetitive sequence of ruminative thoughts. (For a full review of repetitive thought, see Watkins, 2008.)

The findings described above provide a ready explanation for ruminative sequences. For example, an individual is concerned about one or more important goals, such as keeping a relationship or a job, and feels anxious about the many details involved in these, such as the many things necessary for keeping the other person or the boss happy while meeting one’s own needs and desires. With high enough values placed on these goals and subgoals, the individual’s own thought stream provides a continuing source of internal cues that trigger one after another of the individual’s emotional reactions to, and self-generated thoughts about, the various aspects of the goal pursuit. If this concern is more potent emotionally than most of the person’s other concerns, the network of reactivity is likely to keep the individual’s thoughts within the domain of the highly valued concern, as each thought segment triggers another related to the same broad domain or to related domains, with plenty of repetition. Hence one ruminates.

Furthermore, it appears that negative moods tend to skew mind-wandering toward past events (Smallwood and O’Connor, 2011; Smallwood et al., 2011). Once the person has extracted all of the lessons offered by these past events, further repetition is unhelpful, and the rumination is likely mainly to lower mood even further. Individuals with personalities high in negative affect are particularly vulnerable to this pattern. Apart from mulling the past, the individual, under the pressure of prospective loss or failure, may already be somewhat depressed, and the emotional tone of the ruminative sequences is likely to deepen that depression even further (Nolen-Hoeksema et al., 2008).

It is important not to confuse these findings with an impression sown by the title and summary of an article by Killingsworth and Gilbert (2010), that mind-wandering *as such* lowers mood. That is not what their data actually showed. Their participants rated 42.5% of their mind-wandering episodes as about something “pleasant,” with mood then averaging slightly above the overall mood average, roughly equaling mood when not mind-wandering. They rated 31% of the remaining mind-wandering episodes as about something “neutral,” with average mood slightly below overall average but above the mood scale’s midpoint. Participants rated mood as sharply below overall average and below the scale midpoint only during the 26.5% of mind-wandering samples that they characterized as about something “unpleasant.” Thus, only particular thought content, not mind-wandering *as such*, was associated with substantially lowered mood (cf. also Stawarczyk et al., 2012).

A similar result emerged from a conventional questionnaire study (Mar et al., 2012) that found life satisfaction to be negatively correlated with self-reported patterns of daydreaming about inaccessible people (for example, out of the person’s past or strangers) but positively correlated with daydreaming about people with whom the daydreamer was currently close. That is, the association of daydreaming patterns with affect varied (significantly but weakly) with daydream content. There was no consistent association with daydreaming frequency as such.

Killingsworth and Gilbert (2010) also performed time-lag analyses whose results found lower mood in samples obtained after a mind-wandering episode than after a non-mind-wandering episode, but these episodes were on average hours apart, sometimes separated by a night, which suggests that something other than the fact of individual mind-wandering episodes accounted for this result. Their finding is also inconsistent with the results of another investigation that found no overall effect of mind-wandering on mood 15 min later (Poerio, 2012).

## PRINCIPLE 3: CONDUCIVE CIRCUMSTANCES INDUCE GOAL-DIRECTED ACTION

When the individual is in a situation conducive to making progress toward attaining a goal, the response to goal cues takes the form of actions or operant mental acts that advance the goal pursuit. This idea is intuitively obvious, but its elaborations become complicated. Whether a person views a situation as conducive to pursuing a particular goal depends on a decision process that takes into account the anticipated relative gains and losses arising from a particular course of action in comparison with alternative courses of action possible in that situation. An elaborate research area has grown up around this decision process, which one can generally subsume under *Expectancy X Value theory* in psychology and *Expected Utility theory* in economics. This theory, together with recent neuroscientific findings that support it, is briefly reviewed elsewhere (Klinger and Cox, 2011).

If the individual becomes actively operant in pursuing the goal, the situation is transformed into one similar to experimental task activity. Mind-wandering is then typically reduced, and activity in the default-mode network is attenuated, a finding that originally arose out of experimental manipulations leading to the discovery of that network as one whose activity rises spontaneously and regularly in the absence of work on a task (a “resting state”; Raichle et al., 2001).

However, to clarify the relation of the default-mode network to goal-directed action, this network becomes active more broadly than simply during mind-wandering or simply in the absence of operant activity. It is activated during states of unfocused external attention (Stawarczyk et al., 2011b) or perceptual decoupling (Smallwood, 2013a), when attention is turned away from perceptual senses, regardless of whether the turning away is part of a respondent sequence. Moreover, this activity cannot be accounted for by task-related interferences or external distractions (Stawarczyk et al., 2011b). In a compelling experimental dissection, Smallwood et al. (2013b) reported that degree of activation of core regions of the default-mode network (medial prefrontal cortex and posterior cingulate cortex) was associated with faster RTs in simple laboratory tasks that required focusing on memory (the numerical value of a previous stimulus) but was associated with slower RTs when the task required focusing on current stimuli (whether the present number was odd or even). The important point for present purposes is that the activated default-mode network may facilitate an operant response that depends on an internal focus, such as retrieval from memory.

Previous evidence using less focal behavioral activities is consistent with these findings. For example, Spreng et al. (2010) found activation of the default-mode network during autobiographic planning, which entails both retrieval of memories and imagining the future, thus turning attention inward, but not during a visuospatial planning exercise, an adaptation of the Tower of London puzzle, in which attention is turned outward. Earlier, a meta-analysis by Spreng et al. (2009) had found active default-mode components during autobiographic memories, prospection (imagining the future), navigation (imagining one’s location and how to move within it), and theory of mind (taking another person’s perspective). These results clearly suggest that the default-mode network is activated during a variety of perceptually decoupled mental activities. It thus appears that although the respondent components of mind-wandering may depend on the default-mode network, they are far from the only activity supported by that network.

When tasks make large demands on processing, the necessary shift of mental resources from the default-mode network to executive regions is harder to attain with advancing age (Persson et al., 2007). Mind-wandering activity is also modulated in accordance with the factors described below under Principle 5.

To sum up this section, under circumstances conducive to doing something about one’s goals, mind-wandering declines but the default-mode network on which it depends remains active in relation to inner-focused mental activities, such as retrieval from memory or imagining a future scenario. The operant thoughts and actions that replace mind-wandering are, of course, directed at the individual’s goals. Some of these may be intrinsically satisfying ultimate goals, but others may have been imposed on the individual by the need to satisfy other individuals or circumstances that wield some control over access to the person’s intrinsically satisfying goals, such as a partner or a war.

## PRINCIPLE 4: WHEN CIRCUMSTANCES ARE UNFAVORABLE FOR GOAL-DIRECTED OPERANT BEHAVIOR, ACTIVITY BECOMES LARGELY MENTAL, AS IN MIND-WANDERING. WHAT FOR?

When circumstances are unfavorable for goal-directed operant behavior, whether in action or thought, the response to cues of a goal remains largely mental, as in mind-wandering and dreaming, but still reflects the content of the goal pursuit or thematically associated content. The evidence for the goal-relatedness of such respondent mentation is discussed above under Principle 1 (e.g., Klinger, 1978; Hoelscher et al., 1981; Nikles et al., 1998; Andrews-Hanna et al., 2010a; Baird et al., 2011; Stawarczyk et al., 2011a; Schacter, 2012).

### ZONING OUT: MIND-WANDERING WITH BLANK META-AWARENESS

Although such respondent mental content is generally conscious, in the sense that it is at least partly reportable in response to sampling probes, memory for it is often short (median of 5 s per content segment; Klinger, 1978) and it may occur without current awareness. For example, when people were asked to indicate whenever they were aware of having mind-wandered, those reports were much less frequent than the mind-wandering that was found to take place with experience-sampling probes (Schooler et al., 2011). Furthermore, extent of activation in various brain regions of the default-mode network differs somewhat according to whether individuals are aware or unaware of their mind-wandering.

One reason that people mind-wander much more than they are aware of doing may be that mind-wandering and the meta-awareness of doing so engage some of the same brain structures, such as the anterior prefrontal cortex, so that when occupied by mind-wandering these structures cannot also create awareness of the mind-wandering (Schooler et al., 2011). Thus, energy goes into mind-wandering without the mind-wanderer being aware that it is going on.

### BENEFITS OF MIND-WANDERING

Why would our species have evolved such extensive mind-wandering? Actually, this state of not thinking directedly appears to confer a number of benefits for cognitive functioning (Christoff et al., 2011). This section reviews evidence regarding its functions.

### PLANNING FOR GOAL PURSUITS

First among these is the role of mind-wandering states in advancing people toward their goals. Thus, thought samples indicate that the content of mind-wandering includes planning elements, which strongly suggests it fulfills a planning function (Baird et al., 2011; Stawarczyk et al., 2011a), even though its components are spontaneous and fragmentary.

### CREATIVE PROBLEM-SOLVING

Second, mind-wandering appears to promote creative problem-solving that is productive toward attaining one’s goals. There are numerous anecdotal reports of important creative insights attained during states that foster mind-wandering (e.g., Klinger, 1990; Singer, 2009). Early on, Singer and Schonbar (1961) had found that a psychometric, self-report measure of how much graduate students in education daydreamed correlated 0.48 with the degree of creativity the students displayed in their account of an “actual daydream” and in a “spontaneous, original story” that they wrote. Reviewing the challenges one faces, as in mind-wandering, promotes the incubation of creative problem-solving in a way that improves subsequent performance. Thus, interposing an opportunity for mind-wandering (during an undemanding task) between two administrations of Unusual Uses problems leads to better subsequent performance in solving those problems on a second try than after interposing a demanding task that discourages mind-wandering (Baird et al., 2012).

Consistent with these findings, the brain regions of the default-mode network substantially overlap those that come into play in the early stages of creative thinking about something, such as the medial prefrontal cortex. However, creative thinking also eventually requires evaluation of one’s ideas, which involves other regions, such as the lateral prefrontal cortex (Christoff et al., 2011).

### MIND-WANDERING AND DELAY-DISCOUNTING

There are further likely benefits of mind-wandering. It appears that people whose minds wander more than others are also likely to display more patience with receiving rewards and hence make better decisions. Smallwood et al. (2013a) measured mind-wandering with probes for TUTs during an undemanding or a demanding task. Subsequently participants engaged in a “delay-discounting” task. Delay discounting measures the extent to which people settle for smaller immediate rewards rather than wait for larger, delayed rewards. For example, in the study by Smallwood et al. (2013a), participants were given a choice of receiving €10 immediately or a larger reward (ranging from €12 to €50) at from 1 to 180 days later. Considering only the undemanding task, which permitted considerable mind-wandering, those whose minds wandered more than others also were more likely to choose one of the larger but later rewards than the small immediate reward.

In this correlational design, it is impossible to establish causal direction. Does tending toward more mind-wandering provide more opportunity for reflection or more insulation from distracting external stimuli, as Smallwood et al. (2013a) suggest, thereby leading to sounder decisions? Or does a more restrained decision process or greater trust elicit more mind-wandering? Or are both attributes attributable to some more fundamental property of personality? In any event, mind-wandering is here associated with making sounder choices.

### MEMORY CONSOLIDATION DURING MIND-WANDERING

Finally, there may be a memory consolidation benefit of “off-line” waking thinking that is similar to, although weaker than, the well-documented memory consolidation that takes place in sleep. For example, Ellenbogen et al. (2007) trained participants on the alleged rank order of six stimuli that were composed of different color patterns (e.g., Pattern B > Pattern C). The training consisted of learning the ordinal relationships between pairs of these stimuli that were of adjacent ranks. Later, the participants were given unanticipated tests of ordinal relationships between stimuli that were different by two ranks (“one degree of separation”) or three ranks (“two degrees of separation”). This task required participants to exercise inference – to extrapolate from what they had learned during their training experience. With only a 20 min interval after training, participants had little ability to perform this extrapolation task. After 12 or 24 h, however, their performance improved considerably. For the 12-h groups, improvement was roughly similar for inferences across one degree of separation regardless of whether the time interval included a night’s sleep. However, participants who slept during part of the assigned overnight 12-h interval performed better on inferences across two degrees of separation than those who had been assigned the daytime 12-h interval, who presumably stayed awake. It appears that for the awake participants the opportunities for undirected, respondent thought such as mind-wandering fostered the consolidation process, although not as well as in sleep. Christoff et al. (2011) provide an extensive review of the relevant literature and conclude that “recent findings suggest that the off-line processing that occurs during periods of rest is associated with the kind of memory consolidation processes that occur during sleep” (p. 264).

### BENEFITS OF MIND-WANDERING DEPEND ON ITS CONTENTS

The way in which a person’s respondent mentation approaches one’s challenges appears to make an important difference in goal-attainment. Thus Gabriele Oettingen and her colleagues (e.g., Oettingen et al., 2001; Oettingen and Mayer, 2002) undertook an extensive series of studies on how features of fantasies relate to or influence subsequent reactions in imaginary or actual goal pursuits. These studies employed a variety of goals, such as finding employment, study abroad, improved academic performance, and interpersonal relationships. In some studies, participants wrote down fantasy elements and in others their fantasies remained purely mental. In both correlational and experimental designs the results established that, relative to people’s expectancies of success, fantasizing about both the motivating pleasant features of anticipated goal attainment and also the practical steps and potential obstacles on the way to goal attainment led to greater commitment to their goals and better performance than fantasizing about only the positives or only the negatives of their goal pursuits.

The fantasies in these studies were induced by the researchers’ instructions, including in some studies experimentally varied instructions, and in that way they differed from internally generated spontaneous thoughts, as in mind-wandering. They nevertheless represent participants’ self-generated content. It is not unreasonable to extrapolate, subject to future confirmation, that insofar as the prospective fantasies of mind-wandering contain elements that promote planning, people are at an advantage in attaining their corresponding goals.

## PRINCIPLE 5: MIND-WANDERING IS LIKELY TO THE EXTENT THAT ONGOING ACTIVITIES LEAVE SOME MENTAL RESOURCES UNMOBILIZED. WHAT CONDITIONS GOVERN IT?

The more an individual engages in mind-wandering, the greater, presumably, will be the representation of the person’s broad panoply of goals in his or her thought stream. The factors that determine the amount of mind-wandering are therefore relevant to determining the contents of waking thought. Hence the importance of Principle 5: spontaneous-seeming respondent responses as in mind-wandering are more likely (1) the more that an individual is momentarily mentally unoccupied with ongoing tasks, or (2) occupied with easy tasks that place fewer demands for operant resources, and (3) the less that is at stake for the person in an ongoing activity. Also, (4) focused perceptual activity, such as scanning a room to find someone, temporarily suppresses thought. Principle 5 has been well-supported in behavioral research, and recent neurocognitive studies are beginning to provide reasons for it.

The probability of spontaneous-seeming respondent thought is highest during relaxed periods, when the brain’s default network predominates (Mason et al., 2007; Christoff et al., 2009; Andrews-Hanna et al., 2010a), or during sleep. After all, the default network was discovered as a result of researchers in brain-imaging studies observing regularities in brain activity when participants were between their assigned tasks (Raichle et al., 2001), and the mental activity during those task-free episodes was found to be largely mind-wandering (Mason et al., 2007).

### A GROUND-BREAKING INVESTIGATION

An early investigation (Antrobus et al., 1966) that obtained thought reports after brief signal-detection trials established a number of other conditions. First, the rate at which participants had to make judgments and the difficulty of the task (detect a tone of a particular frequency versus detect a change in frequency from the previous tone) both significantly affected reports of task-irrelevant thoughts. That is, the more demanding the task, the less minds wandered.

Second, when the investigators instituted money penalties of differing sizes for missing target signals, higher penalties led to fewer reports of task-irrelevant thoughts (This finding actually applied only to the male participants, for reasons that are unclear).

Third, between sets of such trials in their Experiment 3 the investigators (Antrobus et al., 1966) exposed half of the participants to a radio broadcast of mostly music that was ostensibly interrupted by an (untrue) announcement that the Communist Chinese had just entered the Vietnam war, which was then really in progress, and that U.S. draft boards were calling up all eligible men (untrue at that time). Given the implications of such a development for the male participants, many of whom would have been subject to the military draft, and for participants’ friends and relatives, this procedure clearly instated or elevated a current concern. The effect on the subsequent set of signal-detection trials was clear: compared with the control group, strongly increased rates of reported task-irrelevant thoughts. Later inquiry revealed, unsurprisingly, that many of the TUTs by participants in the experimental condition related to the impact on them of the supposed entry by the Chinese into the war. Here the manipulation was not of what was at stake in participants’ performance but rather what was at stake in external events, which surely relegated task performance to relative irrelevance.

Finally, Antrobus et al. (1966) reported that the rate at which participants reported task-irrelevant thought steadily increased over trials. This was presumably an effect of fatigue, or perhaps also of boredom.

### TASK DIFFICULTY AND MIND-WANDERING

Subsequent research has confirmed these conditions that govern the tendency for minds to wander. One prominent determinant, as in Antrobus et al. (1966), is task difficulty, which can be operationalized as a baseline task of simply fixating attention on a point on a screen versus varying degrees of perceptual load or working memory load.

Giambra (1995) found TUTs more frequent with less demanding vigilance tasks. He reports on other experiments with similar effects, but the difficulty of the reading tasks they used appears not to have affected the frequency of TUTs. Difficulty levels of texts may affect TUTs differently than more controlled, brief task units. Feng et al. (2013) found more TUTs with difficult than with easy texts and also worse comprehension. It may well be that when texts become sufficiently difficult, readers have trouble maintaining the stream of absorbing the text meaningfully, with correspondingly more frequent lapses into mind-wandering.

One way to vary load without changing tasks is by giving participants varying degrees of practice with a task. This is the approach taken by Mason et al. (2007), who found mind-wandering (operationalized as stimulus-independent thoughts [SITs]) most frequent at baseline rest, less frequent with well-practiced tasks, which are to some extent automatized by the practice and hence require less conscious control than novel tasks, and least frequent with novel tasks. Activity levels of the default-mode network varied with the task conditions similarly to SIT rates. These relationships between patterns of brain activity and mind-wandering were confirmed and extended by Christoff et al. (2009), who combined the *f*MRI procedure with simultaneous thought probes during a go/no-go task.

The investigation by Mason et al. (2007) also found significant correlations during well-practiced tasks between *f*MRI readings of activation in six regions of the default-mode network and scores on the daydreaming frequency scale of the Imaginal Processes Inventory (IPI; Singer and Antrobus, 1972), a self-report psychometric measure of the individual’s typical inner experience. With little variation across the six recording sites, the mean of the mean correlations was 0.58; the mean of the peak correlations was 0.72. These strong correlations both validate the daydreaming frequency scale of the IPI and establish the close association of the default-mode network with mind-wandering, which is one form – most likely by far the largest – of daydreaming. (The connection is close but not exclusive. See above the work by Christoff et al. [2009], Smallwood et al. [2013b], Spreng et al. [2010], and others.)

There were a number of partial precedents for these findings: SITs associated with medial prefrontal cortex (McGuire et al., 1996), similarity in activation patterns during rest and semantic retrieval (Binder et al., 1999), increased task difficulty leading (a) to increased deactivation of some regions associated with the default-mode network (McKiernan et al., 2003) and (b) to decreased self-reported TUTs (McKiernan et al., 2006). In each case the investigators suggested that the stimulus-independent and task-unrelated thoughts may represent the kind of self-generated or self-oriented thoughts common in mind-wandering.

Forster and Lavie (2009) varied perceptual load, defined here operationally as, for example, detecting a letter on a screen surrounded by letters that look similar to the target letter, a task that requires close inspection of the various letters, versus letters that look quite different from the target, in which the target letter is easily discriminable. The greater the perceptual load required to perform tasks, the less participants’ minds wandered.

Andrews-Hanna et al. (2010a) have shown that both reports of spontaneous thought and activity in the default-mode network are substantially greater in relatively passive states (fixation on cross-hairs) than during more demanding tasks (detecting subtle visual flickers on a screen). This investigation also found a number of important features linking activity in various brain regions to the content of spontaneous thought. Especially, activity in the medial temporal lobe correlated with default-mode activity when participants reported thinking about something in the past or the future. This leads to the inference that the medial temporal lobe relays long-term memories to the default-mode network’s thought stream.

Teasdale et al. (1995) examined the relationship of three components of working memory to SITs (although stimulus independence and task-unrelatedness are, from the standpoint of self-ratings, virtually uncorrelated dimensions (Klinger and Cox, 1987–1988), both have been used by various researchers to operationalize daydreaming or mind-wandering). One component of working memory is the “phonological loop,” whose relation to SITs was assessed by having participants memorize sequences of digits by speaking them versus having them simply repeat a set sequence of digits without any need to remember them. As compared with quiet conditions, both of these procedures significantly reduced the number of SITs by half or more.

In a second experiment, Teasdale et al. (1995) examined a second component of working memory, the visuospatial sketchpad, operationalized as tapping keys on a keyboard in specified orders. Participants in all three conditions had to decide whether displayed sentences were silly. Compared with a quiet condition, conditions in which they also tapped their fingers more than halved the number of their SITs.

A third experiment investigated the effects of having practiced a task, which consisted either of tracking a point on a pursuit rotor or memorization of digits. Practice permits some automatization of task behavior and hence relieves the need for conscious control. There were fewer than half as many SITs while performing novel as compared with practiced tasks.

Finally, in a fourth experiment, Teasdale et al. (1995) examined the role of central executive resources in frequency of SITs. They did this not by counting SITs but by assigning a task of continuously generating random numbers, which were recorded, and then examining lapses from randomness in the 20 numbers generated before a thought probe. They then compared these indices of randomness according to whether the probe elicited a SIT or a report of “not thought” (NT). On the assumptions that generating random numbers is difficult enough to make significant demands on central executive processes, and that central executive resources are necessary for producing SITs, Teasdale et al. (1995) hypothesized that lapses from randomness would be more frequent before SITs than before NTs. They confirmed this hypothesis, with a difference of a bit less than half a standard deviation.

Teasdale et al. (1995) concluded that SITs require central executive resources, and that the suppression of SITs during the various tasks results from competition for those resources. This formulation is consistent with the generalization that mind-wandering occurs less often during more demanding tasks. What it does not explain is why, in the absence of task activity, the brain automatically reverts to the default-mode network, and why mental activity automatically reverts to mind-wandering, as the baseline, default states of brain and mind.

McKiernan et al. (2003) varied three dimensions of task difficulty: stimulus presentation rate, perceptual discriminability, and short-term memory load, each at three levels that were considered easy, moderately difficult, and difficult. The difficulty levels were reflected in corresponding differences in accuracy and time on target, thus verifying the manipulation of difficulty. In all but one of the tested regions, increased difficulty led to increased deactivation of some regions subsequently associated with the default-mode network. Even the easiest conditions produced some significant deactivation. McKiernan et al. (2003) attribute this effect to an automatic shift of brain resources from the cognitive processes active at “rest” to those necessary to perform the assigned tasks.

Besides the observations that mind-wandering was more frequent during relaxed states or work on undemanding tasks, under these conditions self-generated thoughts were more often focused on the future than on the past (Klinger and Cox, 1987–1988; Smallwood et al., 2009b, Smallwood et al., 2011; Baird et al., 2011). Under more demanding conditions, this prospective bias disappeared (Smallwood et al., 2009b). Also, working memory capacity has been reported inversely associated with everyday mind-wandering during resource-demanding activities (Kane et al., 2007), positively related to proportionally prospective mind-wandering during an undemanding task (Baird et al., 2011), but unrelated to proportionally prospective mind-wandering during demanding tasks (McVay et al., 2013). In the latter condition, perhaps the drain on working memory saps its availability for building scenarios of the future.

### MOTIVATION – WHAT THE PERSON HAS AT STAKE IN PAYING CLOSE ATTENTION

With regard to the effect on mind-wandering of what people have at stake, Kool and Botvinick (2012) examined the amount of time participants spent on a demanding or an undemanding task in sessions in which they could freely shift from one to the other. Demanding-task trials, which, based on previous evidence, presumably reduced the opportunity for mind-wandering, were rewarded with candy pieces, whereas trials with undemanding tasks were not rewarded at all. Participants were allowed to switch back and forth between the two types of task within sessions. In a second session 1–2 weeks later, some participants were told that their candy “wages” had increased and others were told that they had decreased. The time they spent with demanding tasks decreased after a wage decrease and increased after a wage increase. The fact that these participants chose some unrewarded trials is consistent with having a need for freer mental activity, as in mind-wandering, and the shift toward more unrewarded trials when the wages for rewarded, demanding trials decreased indicates that mind-wandering decreases when there is more at stake in the assigned task.

Mind-wandering has a well-established inverse relationship to a variety of performance measures (e.g., Smallwood et al., 2004; McVay and Kane, 2012a,b; Smallwood, 2013b), including reading comprehension (Smallwood et al., 2003, 2007, 2008; Smallwood and Schooler, 2006; McVay and Kane, 2009; Smallwood, 2011; Mrazek et al., 2012). Unsworth and McMillan (2013) thought-sampled TUTs and obtained laboratory measures of working memory capacity and a number of self-report measures: motivation for the task, interest in the topic, and previous experience with the subject matter. They found that, of these, motivation for the reading task was the strongest predictor (-0.61) of the TUT measure of mind-wandering, and mind-wandering was the strongest predictor (-0.58) of reading comprehension. In combination with the findings of Kool and Botvinick (2012) and the original findings of Antrobus et al. (1966), it is clear that, unsurprisingly, mind-wandering gives way to sufficiently important external demands.

When one speaks of goals, goal neglect, and the interference of TUTs with performance, the reference is usually to experimenter-assigned goals that are likely to be of limited importance to the experimental participants. As Antrobus et al. (1966) found when they levied money penalties for inattention (missing target signals), incentives affect TUT rates. It would be interesting to investigate the relation of TUTs to working memory capacity or executive attention, and to task performance, under higher-incentive conditions. Would high-TUT participants then be equally disadvantaged as they have been in the investigations reported hitherto?

### EFFECT OF TASK OR ACTIVITY DURATION

As Antrobus et al. (1966) found, the incidence of TUTs increased with task duration. Smallwood et al. (2002–2003) reported a similar effect for much briefer task periods, interrupting participants after 30, 45, or 60 s with a thought probe. When participants’ tasks were relatively easy to perform, the rate of TUTs increased as a function of duration. This effect did not occur with a task of thinking up words that begin with a particular letter of the alphabet, which requires sustained controlled effort. Other investigators that have reported a similar increase in TUTs as a function of task repetition or duration include Andrews-Hanna et al. (2010a), Stawarczyk et al. (2011a,Experiment 1), McVay and Kane (2012a), and Unsworth and McMillan (2013). None of these investigations obtained direct measures of fatigue or boredom, but it is a reasonable inference that mental fatigue with repeated processing in an operant task might temporarily deplete brain resources, much as has been reported in the literature on ego-depletion (e.g., Baumeister et al., 2000; Baumeister, 2002; Vohs et al., 2013).

### THE TUSSLE BETWEEN MIND-WANDERING AND ATTENTION TO THE EXTERNAL WORLD

Mind-wandering may be the baseline, default mental state, but it is in continuous competition with the need to process what goes on beyond the individual brain. No species could survive without that external attention. McKiernan et al. (2003) have shown with *f*MRI analyses that a variety of task-related variables – stimulus presentation rate, perceptual discriminability, and short-term memory load – in each case contributed to deactivating the default-mode network. They proposed that this happens by diverting attention, which is a limited resource, toward the external task.

Nevertheless, the default-mode network apparently corresponds to the true default mental state in the absence of demands for operant activity by tasks and goals. An essential question is the mechanism for switching between these two attentional orientations. Spreng et al. (2010) proposed a three-network model of how this happens: the default-mode network, the dorsal attention network, and a frontoparietal control network. The first two have “an intrinsic competitive relationship” whereas the third serves “as a cortical mediator linking the two networks in support of goal-directed cognitive processes” (Spreng et al., 2010, p. 303; conceptually extended by Smallwood et al., 2012). That is, Spreng et al.’s (2010) *f*MRI results showed the frontoparietal control network to be activated during both autobiographical (inner-focused) and visuospatial planning (in an adaptation of the Tower of London game). That suggests that the frontoparietal control network plays a key role in the switch back and forth.

### A GREAT DEBATE

For some years now, there has been a lively, constructive debate regarding this switching process. This debate has been most recently summarized by Smallwood (2013a), who has also suggested a resolution among four main viewpoints: the goal theory of current-concerns (e.g., Klinger, 1971, 1975, 1977, 2009; Klinger and Cox, 2011; and the sections above), decoupling from perception (e.g., Smallwood, 2011, 2013a, b), executive control failure (e.g., McVay and Kane, 2009, 2010, 2012a,b), and meta-awareness (Schooler et al., 2011: becoming conscious of one’s mind-wandering, a self-regulatory process). Smallwood proposes separating explanations for the occurrence of mind-wandering from explanations for the process of mind-wandering once it has started. Of the four approaches, only the decoupling approach is relevant to process, in the sense of the continuity and integrity of a thought train. The other three approaches relate to the initiation (“occurrence”) of a mind-wandering thought train. Therefore, decoupling from perception complements rather than conflicts with the other three as an account of mind-wandering.

Smallwood (2013a) is correct in stating that the current concerns/goals theory is about the way in which thought segments start and has little to say about how segments continue or end. Decoupling, on the other hand, addresses a condition necessary to protect an ongoing train of thought from disruption by perceptual demands. Thus, it picks up the theoretical account where current concerns theory leaves off. Furthermore, Smallwood views executive control functions as a “domain-general resource” that is active in organizing and regulating both externally oriented activity and mind-wandering. This view is consistent with the views of such investigators as Teasdale et al. (1995) and Spreng et al. (2010), although the latter view (and that of Smallwood et al., 2012) has evolved from an executive control network to two networks: a dorsal attentional network that is “anticorrelated” with the default-mode network (i.e., when activity levels in one network rise, activity levels in the other fall), and a frontoparietal control network that can join with either one of the other two, depending on task needs, to bestow processing priority. Spreng et al. (2010) focused on mental planning activity. However, as already indicated, mind-wandering, even if somewhat erratic, frequently relates to planning.

The strong evidence for perceptual decoupling raises a further question regarding the processes involved in protecting the integrity of segments of behavior – that is, of relatively integrated response sequences that lead from the decision to act (or the start of a thought train) to the intended endpoint of the sequence (see also Franklin et al., 2013; Smallwood, 2013b). That launching such a sequence instates an inhibition of interruptive factors seems clear. External interruptions of on-going behavior before some logical endpoint or pause “leads to visceral arousal” and emotional upset (Mandler, 1964, p. 163). The entire literature on emotional accompaniments of extinction of operant behaviors attests to this (e.g., Klinger, 1975, 1977). The expectation that accustomed sequences of behavior will end as usual seems ingrained in people from an early age. EEG evidence with event-related potentials indicates that infants as young as nine months react with N400 deflections (negative deflections after 400 ms post-stimulus) when sequences they observe end unexpectedly (Reid et al., 2009). Perceptual decoupling may, accordingly, be part of a more extensive process that protects ongoing behavior (see also Klinger, 1971, 2011, in regard to a meaning-complex theory of response organization; also behavioral chunking, e.g., Perlman et al., 2010).

McVay and Kane’s conceptualization (e.g., Kane and McVay, 2012; McVay and Kane, 2012b), on the other hand, features proactive executive control that keeps people focused on their goal pursuits, from which mind-wandering distracts. Indeed, mind-wandering detracts from performance of many ongoing tasks (Schooler et al., 2011). In an extensive individual differences investigation employing structural equation modeling, TUTs mediated effects of executive attention and working memory capacity on reading comprehension with a coefficient of -0.44 (McVay and Kane, 2012a). Executive control is anticorrelated with the default-mode network (e.g., Buckner et al., 2008); mind-wandering thus represents a transient “failure” of the executive control network rather than a potentially adaptive switch to another network.

Smallwood (2010), 2013a questions this executive-failure view on a number of grounds. First, the content of mind-wandering is internally organized, may be persistent, and hence probably also requires support from an executive control system that supports both attention to a task and the integrity of thought trains that have wandered away from it. Second, mind-wandering interferes with processing of both task-relevant and task-irrelevant cues, suggesting a briefly stable perceptual decoupling that redirects attentional resources to the ongoing stream of thought and is to that extent impervious to distraction from external stimuli. Third, in that individual differences study of the relationship between mind-wandering and reading comprehension (McVay and Kane, 2012b), measures of trait attention control and working memory capacity predicted performance, but after controlling for this, TUTs still accounted for an additional 8% of the variance in comprehension errors, thus suggesting that mind-wandering affects task performance beyond the role of (executive) attention control, presumably because of perceptual decoupling that accompanies mind-wandering (Smallwood, 2013a).

As indicated, perceptual decoupling can be considered applicable to thought process (protecting the integrity of the thought stream), whereas the three other approaches apply to initiation of thought segments. In that case, it would appear that decoupling complements the other three rather than conflicting with them. However, the relationship among them is more complicated than that. Failure of executive control during an ongoing task would represent the beginning of a mind-wandering episode, but reassertion of executive control would require an intrusion on the mind-wandering, breaking through the decoupling shield to refocus on external reality – a recoupling of perception. To that extent, these processes would be in conflict. As a further complication, if, as Smallwood (2013a) suggests, mind-wandering itself requires executive resources, are there then two strands of executive control that come into conflict with each other? Perhaps, but this seems a bit unparsimonious.

One conceptual possibility is that momentary shifts in incentives, in the form of actual or anticipated affect regarding the direction of attention, control the person’s executive processes with corresponding shifts between external perception or action and the fostering of thoughts. The decision function could be similar to that operating in choices among alternative actions (e.g., Knutson et al., 2005; Tobler et al., 2005). Rather than a failure of executive control, this would entail a flexible executive that moves attention to whatever focus appears at the moment optimal not only for task performance but also for brain refreshment and for all of the other goals on the individual’s agenda. The model proposed by Spreng et al. (2010), with a frontoparietal control network that flexibly joins with other networks to selectively empower their functions, seems compatible with this view.

This debate is ongoing. With the latest published arguments barely months old, one can expect further counterarguments and, one would hope, eventual further investigations to clarify the underlying mechanisms for shifts in attention between the external and internal worlds.

### MOTIVATION TO MIND-WANDER

There is reason to believe that mind-wandering is positively valued, that people are motivated to engage in a certain amount of it. The evidence already cited above that mind-wandering increases with task duration could be taken as supportive of it having positive value but could also be interpreted as evidence of weakening executive control (increased ego-depletion). However, the results described above by Kool and Botvinick (2012) are harder to interpret in this way. Given trial-to-trial choice of a highly demanding computer-based task (work) or a task with minimal demands, which would permit extensive mind-wandering, their participants chose to spend some trials in the low-demand task, even though all choices of demanding trials and none of the choices of low-demand trials were rewarded. With increased levels of reward per choice of work trial, but with no accompanying change in task difficulty or duration, the amount of time spent on the high-demand tasks also increased. This latter finding rules out simply mental exhaustion as an explanation for choices of the low-demand trials.

This raises the question: to what extent are people positively motivated to spend time mind-wandering (i.e., under the sway of the default-mode network)? Clearly, people are positively motivated to pursue goals (by definition!), which must limit the amount of time they spend mind-wandering. There is extensive reason to believe that a sense that one’s life is meaningful depends on having attractive, attainable goals and that this is necessary for sustained mental health (Klinger, 1977, 2012). The absence of such goals leads to boredom, depressed moods, excessive entertainment, and substance use. People strive for a balance between active goal pursuit and inner life such as ordinary mind-wandering and imaginative daydreaming, which are themselves goal-related. There are large individual differences in the amount of time that people desire to spend in imaginative daydreaming (Singer and Bonanno, 1990; Singer, 1966, 1975; Bigelsen and Schupak, 2011), some valuing it as a resource for self-amusement and stimulation and others eager to reduce or eliminate it but unable to attain a daydream-free state.

If moving between inner-directed and outward-directed states is a kind of automatic decision process, there must be a switching mechanism that employs some set of criteria for making the switch. It would be interesting to investigate which brain regions are involved and whether they include those identified with decisions using Value × Expectancy criteria, as has been found in previous studies of incentive choices (Knutson et al., 2005; Tobler et al., 2005).

It may be that the human brain is designed to spend some of its time processing and reprocessing its agenda of goals and the person’s experiences that bear on those goals. I am unaware of specific evidence regarding this, but there is suggestive evidence in experiments by Borkovec et al. (1983), who instructed worriers to spend a concentrated half hour of each day worrying. This reduced the self-reported percentages of their remaining hours per day spent worrying. It would be interesting to examine whether time periods immediately following intensely demanding work contain more mind-wandering than during times following relaxed periods or undemanding work. The results described above of increasing mind-wandering with increasing task duration suggest a likely outcome.

In any event, the processing and reprocessing that occurs in mind-wandering acts as a useful continuing examination of the state of one’s goal pursuits and the possibilities and resources for enhancing them, as well as a reminder mechanism that keeps goal-related material fresh in long-term memory and alerts individuals to upcoming goal-related demands for action.

## Conflict of Interest Statement

The author declares that the research was conducted in the absence of any commercial or financial relationships that could be construed as a potential conflict of interest.

## References

[B1] Andrews-Hanna J. (2012). The brain’s default network and its adaptive role in internal mentation. *Neuroscientist* 18 251–270 10.1177/1073858411403316 21677128 PMC3553600

[B2] Andrews-Hanna J. Reidler J. S. Huang C. Buckner R. L. (2010a). Evidence for the default network’s role in spontaneous cognition. *J. Neurophysiol.* 104 322–335 10.1152/jn.00830.2009 20463201 PMC2904225

[B3] Andrews-Hanna J. R. Reidler J. S. Sepulcre J. Poulin R. Buckner R. L. (2010b). Functional-anatomic fractionation of the brain’s default network. *Neuron* 65 550–562 10.1016/j.neuron.2010.02.005 20188659 PMC2848443

[B4] Antrobus J. S. Singer J. L. Greenberg S. (1966). Studies in the stream of consciousness: experimental enhancement and suppression of spontaneous cognitive processes. *Percept. Mot. Skills* 23 399–417 10.2466/pms.1966.23.2.399

[B5] Baird B. Smallwood J. Mrazek M. D. Kam J. W. Y. Franklin M. S. Schooler J. W. (2012). Inspired by distraction: mind wandering facilitates creative incubation. *Psychol. Sci.* 23 1117–1122 10.1177/0956797612446024 22941876

[B6] Baird B. Smallwood J. Schooler J. W. (2011). Back to the future: autobiographical planning and the functionality of mind-wandering. *Conscious. Cogn.* 20 1604–1611 10.1016/j.concog.2011.08.007 21917482

[B7] Baumeister R. F. (2002). Ego depletion and self-control failure: an energy model of the self’s executive function. *Self Identity* 1 129–136 10.1080/152988602317319302

[B8] Baumeister R. F. Muraven M. Tice D. M. (2000). Ego depletion: a resource model of volition, self-regulation, and controlled processing. *Soc. Cogn.* 18 130–150 10.1521/soco.2000.18.2.130

[B9] Beckett S. (1995). “Texts for nothing,” in *The Complete Short Prose of Samuel Beckett, 1929-1989* ed Gontarski S. E. (New York: Grove) 100–154

[B10] Bigelsen J. Schupak C. (2011). Compulsive fantasy: proposed evidence of an under-reported syndrome through a systematic study of 90 self-identified non-normative fantasizers. *Conscious. Cogn.* 20 1634–1648 10.1016/j.concog.2011.08.013 21959201

[B11] Binder J. R. Frost J. A. Hammeke T. A. Bellgowan P. S. Rao S. M. Cox R. W. (1999). Conceptual processing during the conscious resting state. A functional MRI study. * J. Cogn. Neurosci.* 11 80–95 10.1162/089892999563265 9950716

[B12] Bock M. Klinger E. (1986). Interaction of emotion and cognition in word recall. *Psychol. Res.* 48 99-106 10.1007/BF00309323

[B13] Borkovec T. D. Wilkinson L. Folensbee R. Lerman C. (1983). Stimulus control applications to the treatment of worry. *Behav. Res. Ther.* 21 247–251 10.1016/0005-7967(83)90206-1 6615390

[B14] Buckner R. L. Andrews-Hanna J. R. Schacter D. L. (2008). The brain’s default network. *Ann. N. Y. Acad. Sci.* 1124 1–38 10.1196/annals.1440.011 18400922

[B15] Chatard A Selimbegović L. (2011). When self-destructive thoughts flash through the mind: failure to meet standards affects the accessibility of suicide-related thoughts. *J. Pers. Soc. Psychol.* 100 587–605 10.1037/a0022461 21299310

[B16] Christoff K. (2012). Undirected thought: neural determinants and correlates. *Brain Res.* 1428 51–59 10.1016/j.brainres.2011.09.060 22071565

[B17] Christoff K. Gordon A. M. Smallwood J. Smith R. Schooler J. W. (2009). Experience sampling during fMRI reveals default network and executive system contributions to mind wandering. *Proc. Natl. Acad. Sci. U.S.A.* 106 8719–8724 10.1073/pnas.0900234106 19433790 PMC2689035

[B18] Christoff K. Gordon A. M. Smith R. (2011). “The role of spontaneous thought in human cognition,” in *Neuroscience of Decision Making* eds Vartanian O. Mandel D. R. (New York: Psychology Press) 259–284

[B19] Cox W. M. Blount J. P. Rozak A. M. (2000). Alcohol abusers’ and nonabusers’ distraction by alcohol and concern-related stimuli. *Am. J. Drug Alcohol Abuse* 26 489–495 10.1081/ADA-100100258 10976671

[B20] Cox W. M. Fadardi J. S. Pothos E. M. (2006). The addiction-stroop test: theoretical considerations and procedural recommendations. *Psychol. Bull.* 132 443–476 10.1037/0033-2909.132.3.443 16719569

[B21] Cox W. M. Hogan L. M. Kristian M. R. Race J. H. (2002). Alcohol attentional bias as a predictor of alcohol abusers’ treatment outcome. *Drug Alcohol Depend.* 68 237–243 10.1016/S0376-8716(02)00219-3 12393218

[B22] Cox W. M. Klinger E. (2011). “Measuring motivation: the motivational structure questionnaire, personal concerns inventory, and their variants,” in *Handbook of motivational counseling* 2nd Edn eds Cox W. M. Klinger E. (Chichester: Wiley) 161–204 10.1002/9780470979952

[B23] D’Argembeau A. Demblon J. (2012). On the representational systems underlying prospection: evidence from the event-cueing paradigm. *Cognition* 125 160–167 10.1016/j.cognition.2012.07.008 22863411

[B24] D’Argembeau A. Mathy A. (2011). Tracking the construction of episodic future thoughts. *J. Exp. Psychol. Gen.* 140 258–271 10.1037/a0022581 21401291

[B25] Domhoff G. W. (1996). *Finding Meaning in Dreams: A Quantitative Approach*. New York: Plenum Press. 10.1007/978-1-4899-0298-6

[B26] Ellamil M. Dobson C. Beeman M. Christoff K. (2012). Evaluative and generative modes of thought during the creative process. *Neuroimage* 59 1783–1794 10.1016/j.neuroimage.2011.08.008 21854855

[B27] Ellenbogen J. M. Hu P. T. Payne J. D. Titone D. Walker M. P. (2007). Human relational memory requires time and sleep. *Proc. Natl. Acad. Sci. U.S.A.* 104 7723–7728 10.1073/pnas.0700094104 17449637 PMC1863467

[B28] Fadardi J. S. Cox W. M. (2008). Alcohol-attentional bias and motivational structure independently predict alcohol consumption in social drinkers. *Drug Alcohol Depend.* 97 247–256 10.1016/j.drugalcdep.2008.03.027 18502594

[B29] Fadardi J. S. Cox W. M. (2009). Reversing the sequence: reducing alcohol consumption by overcoming alcohol attentional bias. *Drug Alcohol Depend.* 101 137–145 10.1016/j.drugalcdep.2008.11.015 19193499

[B30] Feng S. D’Mello S. Graesser A. C. (2013). Mind wandering while reading easy and difficult texts. *Psychon.* Bull. Rev. 3 586–592 10.3758/s13423-012-0367-y 23288660

[B31] Forster S. Lavie N. (2009). Harnessing the wandering mind: the role of perceptual load. *Cognition* 111 345–355 10.1016/j.cognition.2009.02.006 19327760 PMC2706319

[B32] Foulkes D. Fleisher S. (1975). Mental activity in relaxed wakefulness. *J. Abnorm. Psychol.* 84 66–75 10.1037/h0076164 1110274

[B33] Franklin M. S. Mrazek M. D. Broadway J. M. Schooler J. W. (2013). Disentangling decoupling: comment on Smallwood (2013). *Psychol. Bull.* 139 536–541 10.1037/a0030515 23607431

[B34] Franz E. A. (2012). The allocation of attention to learning of goal-directed actions: a cognitive neuroscience framework focusing on the basal ganglia. *Front. Psychol. * 3:535 10.3389/fpsyg.2012.00535 PMC3527823 23267335

[B35] Freud S. (1953). “The relation of the poet to day-dreaming,” in *Collected Papers* Vol. 4 (London: Hogarth), Original work published 1908

[B36] Giambra L. M. (1995). A laboratory method for investigating influences on switching attention to task-unrelated imagery and thought. *Conscious. Cogn.* 4 1–21 10.1006/ccog.1995.1001 7497092

[B37] Gilboa-Schechtman E. Revelle W. Gotlib I. H. (2000). Stroop interference following mood induction: emotionality, mood congruence and concern relevance. *Cognit*. Ther. Res. 24 491–502 10.1023/A:1005517326981

[B38] Hobson J. A. McCarley R. W. (1977). The brain as a dream state generator: an activation-synthesis hypothesis of the dream process. *Am. J. Psychiatry* 134 1335–1348 10.1176/appi.pn.2013.6b36 21570

[B39] Hoelscher T. J. Klinger E. Barta S. G. (1981). Incorporation of concern- and nonconcern-related verbal stimuli into dream content. *J. Abnorm. Psychol.* 49 88-91 10.1037/0021-843X.90.1.88

[B40] Horovitz S. G. Fukunaga M. de Zwart J. A. van Gelderen P. Fulton S. C. Balkin T. J. (2008). Low frequency BOLD fluctuations during resting wakefulness and light sleep: a simultaneous EEG-fMRI study. *Hum. Brain Mapp.* 29 671–682 10.1002/hbm.20428 17598166 PMC6871022

[B41] Ihssen N. Cox W. M. Wiggett A. Fadardi J. S Linden D. E. J. (2011). Differentiating heavy from light drinkers by neural responses to visual alcohol cues and other motivational stimuli. *Cereb. Cortex* 21 1408–1415 10.1093/cercor/bhq220 21045002

[B42] Johnsen B. H. Laberg J. C. Cox W. M. Vaksdal A. Hugdahl K. (1994). Alcoholic subjects’ attentional bias in the processing of alcohol-related words. *Psychol. Addict. Behav.* 8 111–115 10.1037/0893-164X.8.2.111

[B43] Kane M. J. Brown L. H. McVay J. C. Silvia P. J. Myin-Germeys I. Kwapil T. R. (2007). For whom the mind wanders, and when: an experience-sampling study of working memory and executive control in daily life. *Psychol. Sci.* 18 614–621 10.1111/j.1467-9280.2007.01948.x 17614870

[B44] Kane M. J. McVay J. C. (2012). What mind wandering reveals about executive-control abilities and failures. *Curr. Dir. Psychol. Sci.* 21 348–354 10.1177/0963721412454875

[B45] Killingsworth M. A. Gilbert D. T. (2010). A wandering mind is an unhappy mind. *Science* 330 932 10.1126/science.1192439 21071660

[B46] Klinger E. (1971). *Structure and Functions of Fantasy*. New York: Wiley

[B47] Klinger E. (1975). Consequences of commitment to and disengagement from incentives. *Psychol. Rev.* 82 1-25 10.1037/h0076171

[B48] Klinger E. (1977). *Meaning and Void: Inner Experience and The Incentives in People’s Lives*. Minneapolis: University of Minnesota Press

[B49] Klinger E. (1978). “Modes of normal conscious flow,” in *The Stream of Consciousness: Scientific Investigations into the Flow of Human Experience* eds Pope K. S. Singer J. L. (New York: Plenum)

[B50] Klinger E. (1978–79). Dimensions of thought and imagery in normal waking states. *J. Altered States Conscious.* 4 97-113

[B51] Klinger E. (1990). *Daydreaming*. Los Angeles: Tarcher (Putnam)

[B52] Klinger E. (1996). “Emotional influences on cognitive processing, with implications for theories of both,” in *The Psychology of Action: Linking Cognition and Motivation to Behavior* eds Gollwitzer P. Bargh J. A. (New York: Guilford) 168–189

[B53] Klinger E. (2009). “Daydreaming and fantasizing: thought flow and motivation,” in *Handbook of Imagination and Mental Simulation* eds Markman K. D. Klein W. M. P. Suhr J. A. (New York: Psychology Press) 225–239

[B54] Klinger E. (2011). Response organization of mental imagery, evaluation of descriptive experience sampling, and alternatives: a commentary on Hurlburt’s and Schwitzgebel’s (2007) *Describing Inner Experience?* *J. Conscious. Studies* 18 92–101

[B55] Klinger E. (2012). “The search for meaning in evolutionary perspective and its clinical implications,” in *The Human Quest for Meaning: Theories, Research, and Applications* 2nd Edn ed Wong P. T. P. (New York: Routledge) 23–56

[B56] Klinger E. Barta S. G. Maxeiner M. E. (1980). Motivational correlates of thought content frequency and commitment. *J. Pers. Soc. Psychol.* 39 1222-1237 10.1037/h0077724

[B57] Klinger E. Barta S. G. Maxeiner M. E. (1981). “Current concerns: assessing therapeutically relevant motivation,” in *Assessment Strategies for Cognitive-Behavioral Interventions* eds Kendall P. C. Hollon S. D. (New York: Academic Press) 161–196

[B58] Klinger E. Cox W. M. (1987–1988). Dimensions of thought flow in everyday life. *Imagin. Cogn. Pers.* 7 105–128 10.2190/7K24-G343-MTQW-115V

[B59] Klinger E. Cox W. M. (2011). “Motivation and the goal theory of current concerns,” in *Handbook of Motivational Counseling* 2nd Edn eds Cox W. M. Klinger E. (Chichester: Wiley) 3–47

[B60] Knutson B. Taylor J. Kaufman M. Peterson R. Glover G. (2005). Distributed neural representation of expected value. *J. Neurosci.* 25 4806–4812 10.1523/JNEUROSCI.0642-05.2005 15888656 PMC6724773

[B61] Kool W. Botvinick M. (2012). A labor/leisure tradeoff in cognitive control. *J. Exp. Psychol. Gen*. 10.1037/a0031048[Epubaheadofprint]. PMC3739999 23230991

[B62] Kuppens P. Tuerlinckx F. Russell J. A. Barrett L. F. (2012). The relation between valence and arousal in subjective experience. *Psychol. Bull.* 10.1037/a0030811[Epubaheadofprint]. 23231533

[B63] Larsen R. Diener E. (1987). Affect intensity as an individual difference characteristic. *J. Res. Pers.* 21 1–39 10.1016/0092-6566(87)90023-7

[B64] Mandler G. (1964). The interruption of behavior. *Nebr. Symp. Motiv.* 12 163–219

[B65] Mar R. A. Mason M. F. Litvack A. (2012). How daydreaming relates to life satisfaction, loneliness, and social support: the importance of gender and daydream content. *Conscious. Cogn.* 21 401–407 10.1016/j.concog.2011.08.001 22033437

[B66] Mason M. F. Norton M. I. Van Horn J. D. Wegner D. M. Grafton S. T. Macrae C. N. (2007). Wandering minds: the default network and stimulus-independent thought. *Science* 315 393–395 10.1126/science.1131295 17234951 PMC1821121

[B67] Mason M. F. Zhang S. Dyer R. L. (2010). Male susceptibility to attentional capture by power cues. *J. Exp. Soc. Psychol.* 46 482–485 10.1016/j.jesp.2009.12.014

[B68] McGuire P. K. Paulesu E. Frackowiak R. S. Frith C. D. (1996). Brain activity during stimulus independent thought. *Neuroreport* 7 2095–2099 8930966

[B69] McKiernan K. A. D’Angelo B. R. Kaufman J. N. Binder J. R. (2006). Interrupting the “stream of consciousness”: an fMRI investigation. *Neuroimage* 29 1185–1191 10.1016/j.neuroimage.2005.09.030 16269249 PMC1634934

[B70] McKiernan K. A. Kaufman J. N. Kucera-Thompson J. Binder J. R. (2003). A parametric manipulation of factors affecting task-induced deactivation in functional neuroimaging. *J. Cogn. Neurosci.* 15 394–408 10.1162/089892903321593117 12729491

[B71] McVay J. C. Kane M. J. (2009). Conducting the train of thought: working memory capacity, goal neglect, and mind wandering in an executive-control task. *J. Exp. Psychol. Learn. Mem. Cogn.* 35 196–204 10.1037/a0014104 19210090 PMC2750806

[B72] McVay J. C. Kane M. J. (2010). Does mind wandering reflect executive function or executive failure? Comment on Smallwood and Schooler (2006) and Watkins (2008). *Psychol. Bull.* 136 188–197 10.1037/a0018298 20192557 PMC2850105

[B73] McVay J. C. Kane M. J. (2012a). Why does working memory capacity predict variation in reading comprehension? On the influence of mind wandering and executive attention. *J. Exp. Psychol. Gen.* 141 302–320 10.1037/a0025250 21875246 PMC3387280

[B74] McVay J. C. Kane M. J. (2012b). Drifting from slow to “D’oh!”: working memory capacity and mind wandering predict extreme reaction times and executive control errors. *J. Exp. Psychol. Learn. Mem. Cogn.* 38 525–549 10.1037/a0025896 22004270 PMC3395723

[B75] McVay J. C. Unsworth N. McMillan B. D. Kane M. J. (2013). Working memory capacity does not always support future-oriented mind-wandering. *Can. J. Exp. Psychol.* 67 41–50 10.1037/a0031252 23458550

[B76] Mrazek M. D. Smallwood J. Franklin M. S. Chin J. M. Baird B. Schooler J. W. (2012). The role of mind-wandering in measurements of general aptitude. *J. Exp. Psychol. Gen.* 141 788–798 10.1037/a0027968 22468669

[B77] Nikles C. D. II Brecht D. L. Klinger E. Bursell A. L. (1998). The Effects of current-concern- and nonconcern-related waking suggestions on nocturnal dream content. *J. Pers. Soc. Psychol.* 75 242–255 10.1037/0022-3514.75.1.242 9686462

[B78] Nikula R. Klinger E. Larson-Gutman M. K. (1993). Current concerns and electrodermal reactivity: responses to words and thoughts. *J. Pers.* 61 63-84 10.1111/j.1467-6494.1993.tb00279.x

[B79] Nolen-Hoeksema S. Wisco B. E. Lyubomirsky S. (2008). Rethinking rumination. *Pers. Psychol. Sci.* 3 400–424 10.1111/j.1745-6924.2008.00088.x 26158958

[B80] Oettingen G. Mayer D. (2002). The motivating function of thinking about the future: expectations versus fantasies. *J. Pers. Soc. Psychol.* 83 1198–1212 10.1037/0022-3514.83.5.1198 12416922

[B81] Oettingen G. Pak H. Schnetter K. (2001). Self-regulation of goal-setting: turning free fantasies about the future into binding goals. *J. Pers. Soc. Psychol.* 80 736–753 10.1037/0022-3514.80.5.736 11374746

[B82] Perlman A. Pothos E. M. Edwards D. J. Tzelgov J. (2010). Task-relevant chunking in sequence learning. *J. Exp. Psychol. Hum. Percept. Perform.* 36 649–661 10.1037/a0017178 20515195

[B83] Persson J. Lustig C. Nelson J. K. Reuter-Lorenz P. (2007). Age differences in deactivation: a link to cognitive control? *J. Cogn. Neurosci.* 19 1021–1032 10.1162/jocn.2007.19.6.1021 17536972

[B84] Poerio G. L. (2012). *Emotional Costs of the Wandering Mind? An Exploration of Mind-Wandering Characteristics and their Relationship with Negative Affect in Daily Life*. Unpublished Master’s Thesis, University of Sheffield, Sheffield

[B85] Pope K. S. (1977). *The Flow of Consciousness*. Unpublished Ph.D. dissertation, Yale University, New Haven

[B86] Raichle M. E. (2009). A paradigm shift in functional brain imaging. *J. Neurosci.* 29 12729–12734 10.1523/JNEUROSCI.4366-09.2009 19828783 PMC6665302

[B87] Raichle M. E. MacLeod A. M. Snyder A. Z. Powers W. J. Gusnard D. A. Shulman G. L. (2001). A default mode of brain function. *Proc. Natl. Acad. Sci. U.S.A.* 98 676–682 10.1073/pnas.98.2.676 11209064 PMC14647

[B88] Reid V. M. Hoehl S. Grigutsch M. Groendahl A. Parise E. Striano T. (2009). The neural correlates of infant and adult goal prediction: evidence for semantic processing systems. *Dev. Psychol.* 45 620–629 10.1037/a0015209 19413420

[B89] Riemann B. C. McNally R. J. (1995). Cognitive processing of personally-relevant information. *Cogn. Emot.* 9 325–340 10.1080/02699939508408970

[B90] Schacter D. L. (2012). Adaptive constructive processes and the future of memory. *Am. Psychol.* 67 603–613 10.1037/a0029869 23163437 PMC3815569

[B91] Schneider W. (1987). *Ablenkung und Handlungskontrolle: Eine Kognitiv-Motivationale Perspektive*. Unpublished Diploma thesis, University of Bielefeld, Bielefeld

[B92] Schooler J. W. Smallwood J. Christoff K. Handy T. C. Reichle E. D. Sayette M. A. (2011). Meta-awareness, perceptual decoupling and the wandering mind. * Trends Cogn. Sci.* 15 319–326 10.1016/j.tics.2011.05.006 21684189

[B93] Seligman M. E. P. Yellen A. (1987). What is a dream? *Behav. Res. Ther.* 25 1–24 10.1016/0005-7967(87)90110-0 3593158

[B94] Singer J. L. (1966). *Daydreaming: An Introduction to the Experimental Study of Inner Experience*. New York: Crown Publishing Group/Random House

[B95] Singer J. L. (1975). *The Inner World of Daydreaming*. Oxford: Harper & Row

[B96] Singer J. L. (2009). Researching imaginative play and adult consciousness: implications for daily and literary creativity. *Psychol. Aesthet. Creat. Arts* 3 190–199 10.1037/a0016507

[B97] Singer J. L. Antrobus J. (1972). “Daydreaming, imaginal processes and personality: a normative study,” in *The Nature and Function of Imagery* ed. Sheehan P. (New York: Academic Press)

[B98] Singer J. L. Bonanno G. A. (1990). “Personality and private experience: individual variations in consciousness and in attention to subjective phenomena,” in *Handbook of Personality: Theory and Research* ed.Pervin L. A. (New York: Guilford Press)

[B99] Singer J. L. Schonbar R. A. (1961). Correlates of daydreaming: a dimension of self-awareness. *J. Consult. Psychol.* 25 1–6 10.1037/h0048906

[B100] Skinner B. F. (1935). Two types of conditioned reflex and a pseudo-type. *J. Gen. Psychol.* 12 66–77 10.1080/00221309.1935.9920088

[B101] Skinner B. F. (1953). *Science and Human Behavior*. New York: Macmillan

[B102] Smallwood J. (2010). Why the global availability of mind wandering necessitates resource competition: reply to McVay and Kane (2010). *Psychol. Bull.* 136 202–207 10.1037/a0018673

[B103] Smallwood J. (2011). Mind-wandering while reading: attentional decoupling, mindless reading and the cascade model of inattention. *Lang. Linguist. Compass* 5 63–77 10.1111/j.1749-818X.2010.00263.x

[B104] Smallwood J. (2013a). Distinguishing how from why the mind wanders: a process–occurrence framework for self-generated mental activity. *Psychol. Bull.* 139 519–535 10.1037/a0030010 23607430

[B105] Smallwood J. (2013b). Searching for the elements of thought: reply to Franklin, Mrazek, Broadway, and Schooler (2013). *Psychol. Bull.* 139 542–547 10.1037/a0031019 23607432

[B106] Smallwood J. M. Baracaia S. F. Lowe M. Obonsawin M. (2003). Task unrelated thought whilst encoding information. *Conscious. Cogn.* 12 452–484 10.1016/S1053-8100(03)00018-7 12941287

[B107] Smallwood J. Brown K. Baird B. Schooler J. W. (2012). Cooperation between the default mode network and the frontal-parietal network in the production of an internal train of thought. *Brain Res.* 1428 60–70 10.1016/j.brainres.2011.03.072 21466793

[B108] Smallwood J. Fishman D. J. Schooler J. W. (2007). Counting the cost of an absent mind: mind wandering as an underrecognized influence on educational performance. *Psychon. Bull. Rev.* 14 230–236 10.3758/BF03194057 17694906

[B109] Smallwood J. Fitzgerald A. Miles L. K. Phillips L. H. (2009a). Shifting moods, wandering minds: negative moods lead the mind to wander. *Emotion,* 9 271–276 10.1037/a0014855 19348539

[B110] Smallwood J. Nind L O’Connor R. C. (2009b). When is your head at? an exploration of the factors associated with the temporal focus of the wandering mind. *Conscious. Cogn.* 18 118–125 10.1016/j.concog.2008.11.004 19121953

[B111] Smallwood J. McSpadden M. Schooler J. W. (2008). When attention matters: the curious incident of the wandering mind. *Mem. Cognit.* 36 1144–1150 10.3758/MC.36.6.1144 18927032

[B112] Smallwood J. Obonsawin M. Reid H. (2002–2003). The effects of block duration and tasks demands on the experience of task unrelated thought. *Imagin. Cogn. Pers.* 22 13–31 10.2190/TBML-N8JN-W5YB-4L9R

[B113] Smallwood J O’Connor R. C. (2011). Imprisoned by the past: unhappy moods lead to a retrospective bias to mind wandering. *Conscious. Cogn.* 25 1481–1490 10.1080/02699931.2010.545263 21432633

[B114] Smallwood J. O’Connor R. C. Sudberry M. V. Haskell C. Ballantyne C. (2004). The consequences of encoding information on the maintenance of internally generated images and thoughts: the role of meaning complexes. *Conscious. Cogn.* 13 789–820 10.1016/j.concog.2004.07.004 15522632

[B115] Smallwood J. Ruby F. J. M. Singer T. (2013a). Letting go of the present: mind-wandering is associated with reduced delay discounting. *Conscious. Cogn.* 22 1–7 10.1016/j.concog.2012.10.007 23178292

[B116] Smallwood J. Tipper C. Brown K. Baird B. Engen H. Michaels J. R. (2013b). Escaping the here and now: evidence for a role of the default mode network in perceptually decoupled thought. *Neuroimage,* 69 120–125 10.1016/j.neuroimage.2012.12.012 23261640

[B117] Smallwood J. Schooler J. W. (2006). The restless mind. *Psychol. Bull.* 132 946–958 10.1037/0033-2909.132.6.946 17073528

[B118] Smallwood J. Schooler J. W. Turk D. J. Cunningham S. J. Burns P. Macrae C. N. (2011). Self-reflection and the temporal focus of the wandering mind. *Conscious. Cogn.* 20 1120–1126 10.1016/j.concog.2010.12.017 21277803

[B119] Spreng R. N. Mar R. A Kim A. S. N. (2009). The common neural basis of autobiographical memory, prospection, navigation, theory of mind, and the default mode: a quantitative meta-analysis. *J. Cogn. Neurosci.* 21 489–510 10.1162/jocn.2008.21029 18510452

[B120] Spreng R. N. Stevens W. D. Chamberlain J. P. Gilmore A. W. Schacter D. L. (2010). Default network activity, coupled with the frontoparietal control network, supports goal-directed cognition. *Neuroimage * 53 303–317 10.1016/j.neuroimage.2010.06.016 20600998 PMC2914129

[B121] Stawarczyk D. Majerus S. Maj M. Van der Linden M D’Argembeau A. (2011a). Mind-wandering: phenomenology and function as assessed with a novel experience sampling method. *Acta Psychol.* 136 370–381 10.1016/j.actpsy.2011.01.002 21349473

[B122] Stawarczyk D. Majerus S. Maquet P D’Argembeau A. (2011b). Neural correlates of ongoing conscious experience: both task-unrelatedness and stimulus-independence are related to default network activity. *PLoS ONE * 6:e16997 10.1371/journal.pone.0016997 PMC3038939 21347270

[B123] Stawarczyk D. Majerus S. Van der Linden M D’Argembeau A. (2012). Using the daydreaming frequency scale to investigate the relationships between mind-wandering, psychological well-being, and present-moment awareness. *Front. Psychol.* 3:363 10.3389/fpsyg.2012.00363 PMC3457083 23055995

[B124] Teasdale J. D. Dritschel B. H. Taylor M. J. Proctor L. Lloyd C. A. Nimmo-Smith I. (1995). Stimulus-independent thought depends on central executive resources. *Mem. Cognit.* 23 551–559 10.3758/BF03197257 7476241

[B125] Tobler P. N. Fiorillo C. D. Schultz W. (2005). Adaptive coding of reward value by dopamine neurons. *Science* 307 1642–1645 10.1126/science.1105370 15761155

[B126] Unsworth N. McMillan B. D. (2013) Mind Wandering and Reading Comprehension: examining the roles of working memory capacity, interest, motivation, and topic experience. *J. Exp. Psychol. Learn. Mem. Cogn*. 39 832–842 10.1037/a0029669 22905931

[B127] Vohs K. D. Baumeister R. F. Schmeichel B. J. (2013). “Motivation, personal beliefs, and limited resources all contribute to self-control”: Erratum. *J. Exp. Soc. Psychol*. 49 183–183 10.1016/j.jesp.2012.08.006

[B128] Watkins E. R. (2008). Constructive and unconstructive repetitive thought. *Psychol. Bull.* 134 163–206 10.1037/0033-2909.134.2.163 18298268 PMC2672052

[B129] Young J. (1987). *The Role of Selective Attention in the Attitude-Behavior Relationship*. Unpublished Ph.D. dissertation, University of Minnesota, Minnesota

